# Morphine interaction with prasugrel: a double-blind, cross-over trial in healthy volunteers

**DOI:** 10.1007/s00392-015-0927-z

**Published:** 2015-10-22

**Authors:** Eva-Luise Hobl, Birgit Reiter, Christian Schoergenhofer, Michael Schwameis, Ulla Derhaschnig, Irene Marthe Lang, Thomas Stimpfl, Bernd Jilma

**Affiliations:** Department of Clinical Pharmacology, Medical University of Vienna, Waehringer Guertel 18-20, 1090 Vienna, Austria; Clinical Institute of Medical and Chemical Laboratory Diagnostics, Medical University of Vienna, Vienna, Austria; Department of Internal Medicine II, Division of Cardiology, Medical University of Vienna, Vienna, Austria

**Keywords:** Drug interactions, Morphine, Platelet function tests, Prasugrel, Vasodilator-stimulated phosphoprotein

## Abstract

**Background:**

Morphine decreases the concentrations and effects of clopidogrel, which could lead to treatment failure in myocardial infarction.

**Objectives:**

To clarify whether more potent P2Y_12_-inhibitors may provide an effective alternative, we examined drug–drug interactions between morphine and prasugrel.

**Methods:**

Twelve healthy volunteers received 60 mg prasugrel with placebo or 5 mg morphine intravenously in a randomized, double-blind, placebo-controlled, cross-over trial. Pharmacokinetics were determined by liquid chromatography tandem mass spectrometry, and prasugrel effects were measured by platelet function tests.

**Results:**

Morphine neither diminished total drug exposure (AUC), which was the primary endpoint, nor significantly delayed drug absorption of prasugrel. However, morphine reduced maximal plasma concentrations (*C*_max_) of prasugrel active metabolite by 31 % (*p* = 0.019). Morphine slightly, but not significantly, delayed the onset of maximal inhibition of platelet plug formation under high shear rates (30 vs. 20 min). Whole blood aggregation was not influenced.

**Conclusions:**

Although morphine significantly decreases the maximal plasma concentrations of prasugrel active metabolite, it does not diminish its effects on platelets to a clinically relevant degree in healthy volunteers. However, it should be considered that the observed decrease in *C*_max_ of prasugrel active metabolite caused by morphine co-administration may gain relevance in STEMI patients.

*Clinical Trial Registration*: NCT01369186, EUDRA-CT#: 2010-023761-22.

## Introduction

Coronary heart disease is one of the most common causes of death worldwide and numerous publications document the permanently increasing research activity in the field of acute coronary syndromes (ACS) [1–11].

As adenosine-5′-diphosphate (ADP) is one of the primary mediators for platelet aggregation, the administration of P2Y_12_-inhibitors in combination with aspirin is a mainstay in the treatment of patients with acute coronary syndromes [12].

In contrast to the extensive evidence that ADP inhibitors are beneficial in patients suffering from myocardial infarction, such data from randomized controlled trials are lacking for morphine. Interestingly, the use of morphine is associated with higher mortality in patients with non-ST-segment elevation ACS [13]. While this is not a causal proof, there may be a biologically plausible cause–effect relationship: opiates inhibit gastric emptying which delays drug absorption and may decrease peak plasma levels of peroral drugs [14].

Indeed, a recent randomized, controlled trial demonstrated that morphine lowers the plasma levels of clopidogrel as well as its antiplatelet effects [15], which could lead to treatment failure in susceptible patients.

It can be hypothesized that more potent P2Y_12_-inhibitors may provide a more effective alternative to clopidogrel when morphine is given, but their interaction with morphine has to be evaluated. Whereas the prodrug clopidogrel is converted into its active metabolite by cytochrome P450 enzymes in two steps [16, 17], prasugrel is rapidly hydrolyzed by esterases to an intermediate metabolite and requires only one further CYP-dependent oxidation step to generate its active compound [16–18]. This produces more active metabolite [19], reduces the variability in response between patients and leads to a more consistent and stronger inhibition of platelets [20–24]. We therefore hypothesized that the observed negative interaction between morphine and clopidogrel [15] may be partially mitigated when prasugrel is used instead of clopidogrel, and conducted this randomized controlled trial to investigate the effect of morphine on the pharmacodynamics and pharmacokinetics of prasugrel.

## Methods

### Experimental design and blood collection

A double-blind, block-randomized, placebo-controlled, cross-over trial was conducted in accordance with good clinical practice guidelines and the Declaration of Helsinki to evaluate the effects of morphine on the intestinal resorption, pharmacokinetics and pharmacodynamics of prasugrel. The study was approved by the Ethics Committee of the Medical University of Vienna and the Austrian National Competent Authority and registered at ClinicalTrials.gov (NCT01369186); written informed consent was obtained from all healthy subjects (*n* = 12).

Key inclusion criteria were: >18 years of age; non-pregnant; the ability to comprehend the full nature and purpose of the study. Key exclusion criteria were: intake of non-steroidal anti-inflammatory drugs or platelet inhibitors; known coagulation disorders; relevant impairment of renal or hepatic function; chronic infectious diseases (HIV, hepatitis B and C); clinically relevant abnormal laboratory values; and contraindications for prasugrel or morphine.

**Fig. 1:**
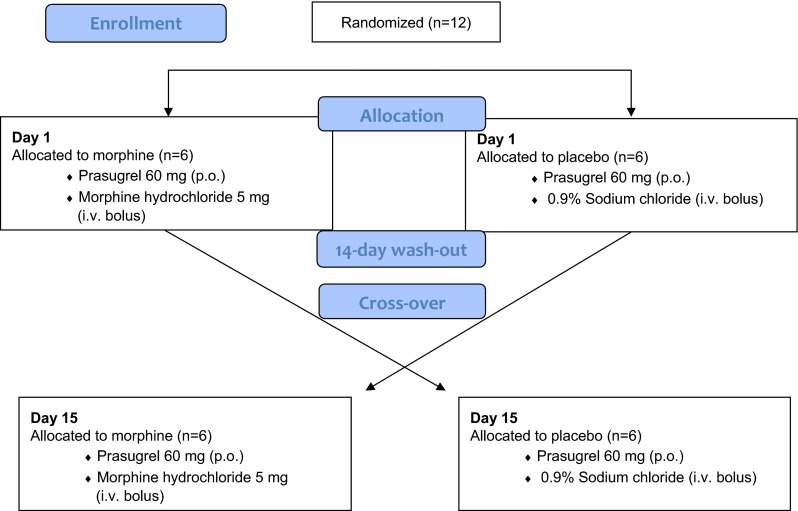
Schematic of trial design

Secretaries conducted randomization by www.randomization.com, and prepared individually sealed opaque envelops. Morphine (5 mg i.v. bolus; Vendal, G.L. Pharma, Lannach, Austria) or placebo (0.9 % NaCl) were prepared by unblinded pharmacists and injected by blinded physicians. A minimum wash-out period of 14 days was chosen (Fig. 1) because it exceeds platelet survival in vivo and because the effect of P2Y_12_-inhibition diminishes within 5 days [25].

After an overnight fast, a loading dose of 60 mg prasugrel (Efient, Eli Lilly, Vienna, Austria) was administered with 250 mL tap water immediately after the injection of placebo or morphine. No food, drink or tobacco was permitted for 4 h.

**Fig. 2:**
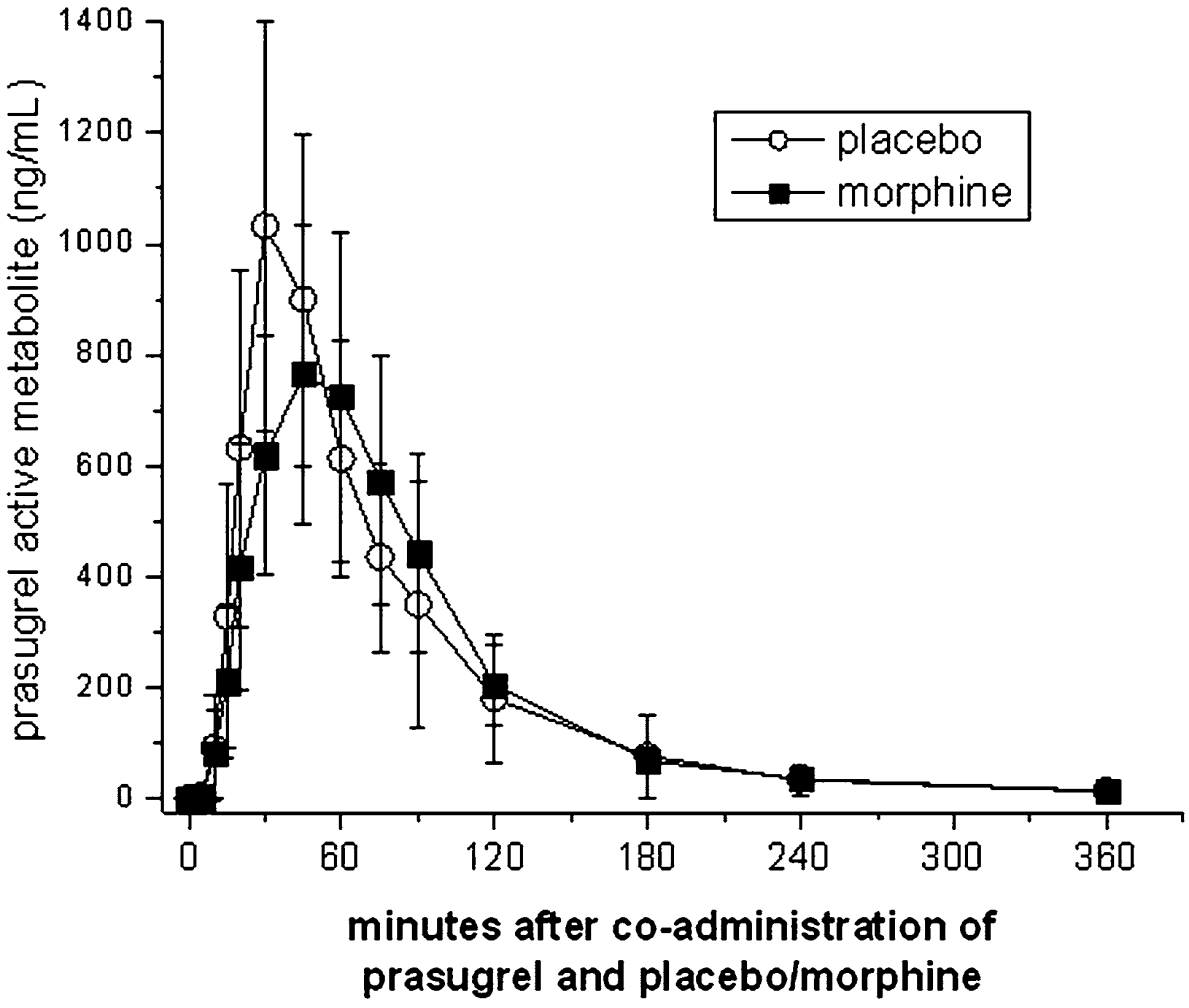
Morphine lowers maximum plasma concentrations of prasugrel active metabolite. Healthy volunteers (*n* = 12) received a 60 mg loading dose concomitantly with a placebo or 5 mg morphine. Data present means ± 95 % CI. *p* values for the comparisons between placebo and morphine: AUC 0.239; *C*_max_ 0.019*; *T*_max_ 0.798 (*indicates significance)

**Fig. 3:**
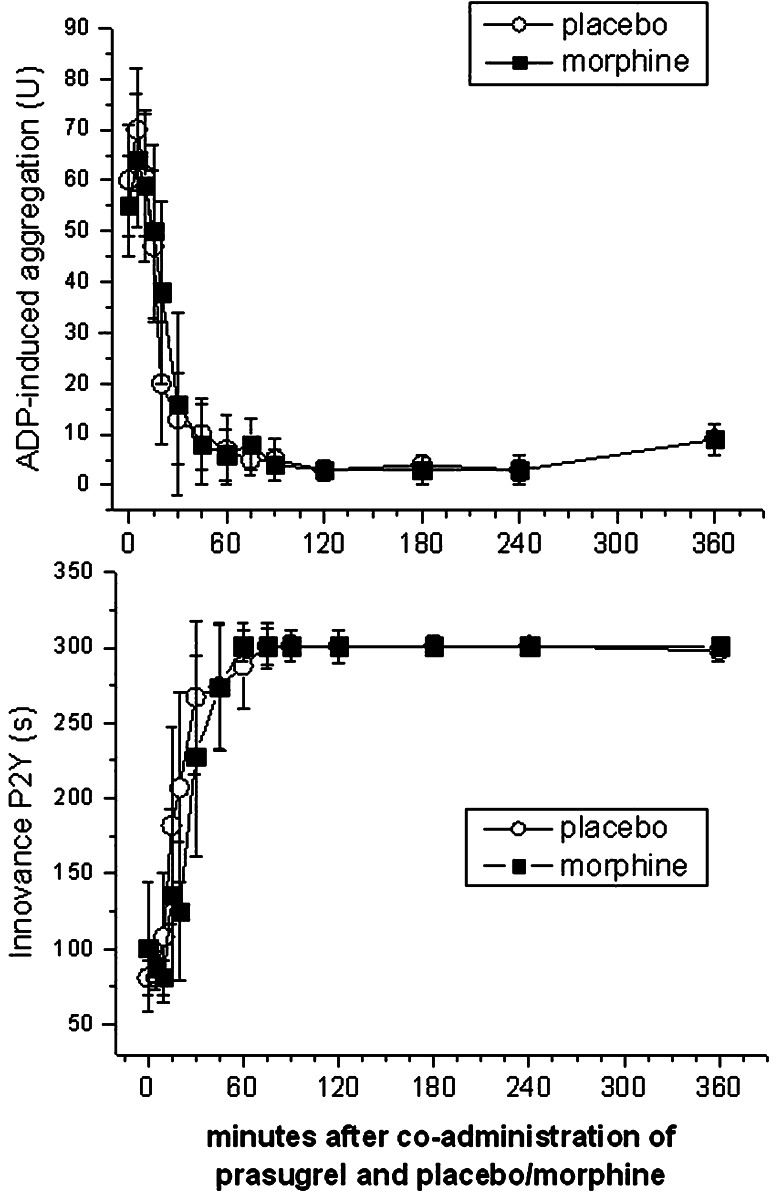
Morphine does not retard or decrease prasugrel effects to a clinically relevant degree. Adenosine diphosphate-induced aggregation was measured by whole blood aggregometry (*n* = 12) and with the P2Y-cartridge of the platelet function analyzer (*n* = 12). Since significance is lost after correction for multiple comparisons, at none of the time points there was a significant difference between placebo and morphine. Data present means ± 95 % CI

**Fig. 4:**
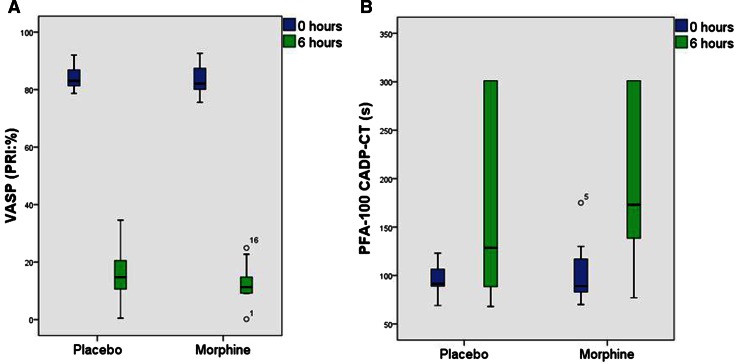
Morphine does not blunt prasugrel effects. *Box- and whisker plot* of the vasodilator-stimulated phosphoprotein (VASP) phosphorylation (**a**) and the collagen adenosine diphosphate closure time (CADP-CT) (**b**) 0 and 6 h after co-administration of prasugrel and placebo/morphine. VASP phosphorylation was measured by flow-cytometry (*n* = 12) and the CADP-CT with the platelet function analyzer (*n* = 12). Data present medians with 25th and 75th percentiles (minimum and maximum)

Blood sampling times for pharmacodynamic and pharmacokinetic evaluations after study drug administration were 5, 10, 15, 20, 30, 45, 60, 75, 90, 120, 180, 240 and 360 min (Figs. 2, 3 and 4). Blood was collected using an i.v. catheter after drawing a waste sample. The analysts were also blinded with regard to the sequence of periods.

### Assessment of pharmacokinetics and pharmacodynamics

Prasugrel effects were measured with the following assays: multiple electrode aggregometry [26], where the intercept of the individual down-slope and the plateau phase was plotted graphically for the area under the curve (AUC) to estimate the onset of the maximum effect, and with the platelet function analyzer under high shear rates [27], where the onset of the maximum effect was defined as the first of three consecutive measurements of >300 s.

To determine the vasodilator-stimulated phosphoprotein (VASP) phosphorylation state of the whole blood, a standardized flow cytometric assay (PLT VASP, BioCytex, Marseille, France) was used, initially described by Schwarz et al. [28]. Blood samples collected in 3.8 % sodium citrate (BD Vacutainer, Becton–Dickinson, Schwechat, Austria) were incubated in vitro with adenosine diphosphate and/or prostaglandin E1 (PGE1) before fixation. For the presentation of data, we used the median fluorescence intensity (MFI) as described earlier [26].

Whole blood aggregation was determined by an impedance aggregometer (Multiple Platelet Function Analyzer/Multiplate Analyzer, Roche, Vienna, Austria). As recommended by the manufacturer, we used hirudin as an anticoagulant. When activated in the test cartridge, platelets attach onto metal electrodes and aggregate, resulting in increased electrical resistance. This change in impedance is recorded for 6 min and is proportional to platelet aggregation. Blood was diluted with saline solution (0.9 %) at a 1:1 ratio and incubated for 3 min. After stirring at 37 °C, analyses were performed using the agonists ADP (6.4 µM) and PGE1 (9.4 µM) (Dynabyte Medical, Munich, Germany). Results were expressed as areas under the curve of the aggregation tracing (AUC). Following the recommendations of the manufacturer and for simpler presentation purposes, we expressed the results as units (U) [29].

Platelet plug formation under high shear rates (5000–6000 s^−1^) was quantified by the platelet function analyzer (PFA-100, Siemens Healthcare Diagnostics, Vienna, Austria). Blood samples were collected in 3.8 % sodium citrate. The PFA-100 measures the time required for occlusion of the aperture by platelet plugs, which is defined as closure time (CT). The maximum duration of a measurement is 300 s. The instrument aspirates a blood sample under constant vacuum from the sample reservoir through a capillary and a microscopic aperture (147 lm) cut into the membrane, which leads to high shear induced platelet plug formation. The membrane is coated with collagen/adenosine diphosphate (CADP). The P2Y-innovance cartridge, which is more sensitive to prasugrel, was used to follow the evolution of platelet inhibition under high shear rates over time. In addition, the CADP-CT was measured at baseline and after 6 h (reference range 65–120 s) [30, 31].

Plasma concentrations of prasugrel active metabolite were determined by liquid chromatography tandem mass spectrometry. Blood samples (6 mL) were collected in pre-cooled EDTA tubes (BD Vacutainer, Becton–Dickinson, Schwechat, Austria). The blood samples were gently inverted and centrifuged within 30 min at 1400×*g* (15 min, 2–8 °C) to separate the plasma. Aliquots were stored at −80 °C and analyzed within 3 months. The applied system consisted of a Symbiosis ALIAS chromatographic system (Spark Holland B.V., Emmen, Netherlands) and an AB Sciex detector (QTRAP 5500, AB Sciex, Framingham, MA, USA). A published procedure [32] was modified to perform the analyses.

### Statistical analysis

The sample size calculation was based on our previously performed drug–drug interaction trial with clopidogrel [15]. Assuming a drop-out rate of 10 %, we calculated that we would need 12 subjects in a cross-over design to achieve 90 % power (α = 0.05).

Pharmacokinetic calculations were made using Kinetica 2000^®^ version 3.0 (InnaPhase Corporation, Philadelphia, Pennsylvania). As usual for drug interaction studies, the primary pharmacokinetic outcome variable was the AUC of prasugrel active metabolite; all other comparisons were considered secondary.

Data are presented as means for demographic data, and medians for outcome variables in the text. Changes in all outcome variables were compared by non-parametric Wilcoxon signed-rank tests, accounting for the skewed distributions of the measurements. To assess the robustness of results, a mixed-model was fitted to test for period and carry-over effects for the outcome variables, which showed a significant effect on these analyses.

Statistical calculations were performed using commercially available software (IBM SPSS Statistics^®^, Version 20, and SAS^®^, Version 9.3). In all cases, two-sided *p* values <0.05 were considered significant.

## Results

### Demographic characteristics of subjects and adverse events

Healthy volunteers (seven males, five females; 11 Caucasian, one Asian) were 30 ± 10 years of age, had 69 ± 11 kg, and a body mass index of 23 ± 3 kg/m^2^. No clinically relevant adverse events were observed after morphine injection, in particular no vomiting occurred.

### Pharmacokinetics

Morphine did not significantly reduce the total exposure as measured by the AUC_0–n_ (69,573 vs. 65,991 ng × h/mL, *p* = 0.239), which was the primary study endpoint; i.e., the mean intra-individual difference was only 5 %. Morphine injection did not influence the time of maximal plasma concentrations of prasugrel active metabolite (30 vs. 38 min, *p* = 0.798), corresponding to a mean intra-individual difference of only 1 min. Similarly, However, morphine reduced the *C*_max_ of prasugrel active metabolite by 31 % from 1388 to 951 ng/mL (*p* = 0.019) (Table 1; Fig. 2).

**Table 1:** Pharmacokinetic parameters of prasugrel active metabolite after a loading dose of 60 mg

Parameter	Placebo	Morphine	*p* value
AUC_0–*n*_ (ng × h/mL)	69,573 (58,898–92,962)	65,991 (50,216–88,390)	0.239
*C*_max_ (ng/mL)	1388 (1116–1507)	951 (821–1106)	0.019
*T*_max_ (min)	30 (30–45)	38 (30–60)	0.798

Values are medians (±interquartile range); *n* = 12

### Pharmacodynamics

Whole blood aggregation was not influenced by morphine co-administration, showing maximum inhibition on average 30–45 min after prasugrel intake (Fig. 3). Only one subject in each period needed 60–75 min to reach near maximal platelet aggregation.

Co-administration of morphine slightly delayed the maximal inhibition of platelet plug formation under high shear rates (30 vs. 20 min), but significance is lost after correction for multiple comparisons (Fig. 3).

No differences in the VASP phosphorylation state (Fig. 4a) and in the conventional collagen/ADP induced closure times (CADP-CT) (Fig. 4b) were observed between periods 6 h after morphine injection.

In general, prasugrel reduced the median platelet reactivity index in the VASP assay within 6 h from a median of 83 to 15 % under placebo, and from 82 to 11 % after morphine (for both periods: *p* < 0.001, Fig. 4a).

Prasugrel intake prolonged the CADP-CT 6 h after intake from a median of 92–129 s under placebo and from 89 to 173 s when morphine was co-administered (for both periods: *p* < 0.001, Fig. 4b).

No significant carry-over or period effects were observed for any of the outcome parameters.

## Discussion

As prasugrel is a more potent P2Y_12_-inhibitor than clopidogrel [20, 21] we hypothesized that it has the potential to overcome the pharmacodynamic problems of the recently described clopidogrel–morphine interaction [15] and characterized the PK/PD interaction between morphine and prasugrel.

Morphine did not reduce the total drug exposure as measured by the AUC_0–n_, which was the primary study endpoint (Table 1; Fig. 2). A 25 % change in AUC is considered a mild to moderate interaction according to the classification of the US Food and Drug Administration [33]. Our trial provided a power of 100 % to exclude even such a mild interaction.

Similarly, morphine injection did not retard the *T*_max_ of the prasugrel active metabolite (Table 1; Fig. 2), although we achieved a power of 92 % to detect a 1 h delay. This is in contrast to an average 2 h delay in the *T*_max_ of clopidogrel active metabolite after morphine injection [15]. However, morphine co-administration reduced the maximum plasma concentrations of prasugrel active metabolite by 31 % (with a power of 92 %) (Table 1; Fig. 2). This could be clinically relevant if morphine reduced the pharmacodynamic effects of prasugrel. Even though co-administration of morphine resulted in a 10 min delay in reaching maximal inhibition of platelet plug formation under high shear rates (30 vs. 20 min), significance is lost after correction for multiple comparisons. Prasugrel maximally inhibited platelet function 30–45 min after intake in both treatment periods (Fig. 3).

In addition, the other platelet function tests such as whole blood aggregation, the VASP phosphorylation state or collagen/ADP induced closure times were not influenced by morphine (Figs. 3, 4). As all platelet assays consistently refute a significant impact of morphine on prasugrel effects, we were not able to prove a pharmacodynamic interaction of morphine with a 60 mg loading dose of prasugrel. The currently authorized loading dose of 60 mg prasugrel appears adequate to inhibit platelet function (Figs. 3, 4) in healthy volunteers, even when the maximal plasma concentrations of prasugrel active metabolite are reduced by ~30 % (Table 1; Fig. 2).

The active metabolites of prasugrel and clopidogrel have comparable anti-platelet activity in vitro. Hence, the higher in vivo potency of prasugrel reflects the more efficient generation of its active metabolite [34]. This is also demonstrated by the current pharmacokinetic data, which show that maximal plasma concentrations and exposure to the active metabolite of prasugrel (Table 1) are several-fold higher than that of clopidogrel [15] despite the tenfold higher clopidogrel loading dose.

One limitation of the current randomized trial is the investigation of healthy volunteers rather than STEMI patients, whose gastrointestinal absorption may be further compromised, e.g., by reduced splanchnic blood flow [35]. However, our cross-over design is considered most adequate for the detection of drug–drug interactions [33]. The resulting low intra-individual variability in such a cross-over design yields exclusively high power to exclude even mild interactions (100 % power for AUC) in a relatively limited sample size.

In conclusion, morphine co-administration moderately decreases the maximal plasma concentrations of prasugrel active metabolite but does not inhibit its effects on platelets to a clinically relevant degree in healthy volunteers.

Therefore, a 60 mg loading dose of prasugrel seems to be effective when morphine is given, but it should be considered that the observed changes in the maximum plasma concentrations of prasugrel active metabolite caused by morphine co-administration may gain relevance in STEMI patients.
